# Giant peritoneal loose body in the pelvic cavity confirmed by laparoscopic exploration: a case report and review of the literature

**DOI:** 10.1186/s12957-015-0539-0

**Published:** 2015-03-24

**Authors:** Hong Zhang, Yun-zhi Ling, Ming-ming Cui, Zhi-xiu Xia, Yong Feng, Chun-sheng Chen

**Affiliations:** Department of Colorectal Surgery, Shengjing Hospital, China Medical University, No. 36 SanHao Street, Heping District, Shenyang, Liaoning 110004 China

## Abstract

A 51-year-old previously healthy male underwent a routine medical examination. Computed tomography and ultrasonography showed an oval-shaped mass that was about 50 × 40 mm in size in the left iliac fossa. Prior to surgery, the lesion was suspected to be a teratoma with core calcification or stromal tumor derived from the rectosigmoid colon. During the procedure, a yellow-white, egg-shaped mass was discovered that was completely free from the pelvic cavity in front of the rectum. The giant, peritoneal loose body was taken out through the enlarged port site. Histological examination showed that the mass consisted of well-circumscribed, unencapsulated, paucicellular tissue, with an obviously hyalinized fibrosclerotic center. A giant peritoneal body is extremely rare. We report such a case and review previously published literature.

## Background

Peritoneal loose bodies are rare. They are usually found at laparotomy or autopsy by accident. In most cases, these bodies are derived from appendix epiploica. The most common size of loose bodies is about from 5 to 20 mm in diameter. Occasionally, they grow to larger than 50 mm by absorbing protein from peritoneal serum [1,2]. We report a case of a giant peritoneal loose body measuring 50 × 40 × 40 mm in the pelvic cavity which happened in a 51-year-old man and confirmed by laparoscopic exploration.

## Case presentation

**Figure 1:**
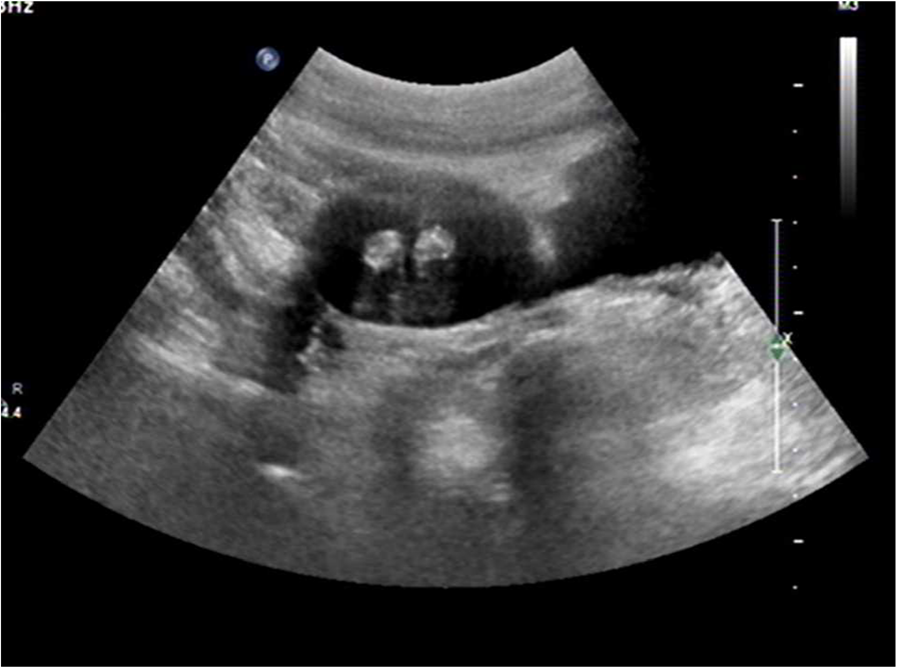
**Two-dimensional ultrasound imaging showed a solid mass with clear boundary.** It was hypoechoic with hyperechoic spots in the central part.

**Figure 2:**
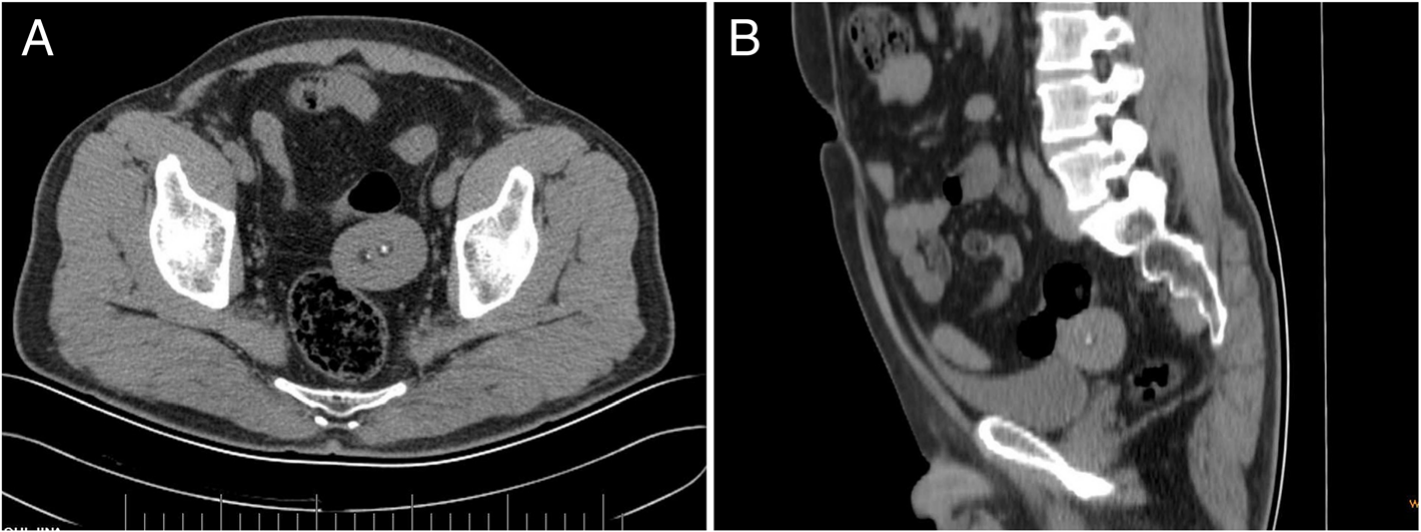
**Abdominal computed tomography findings. (A)** Axial image demonstrated a low-density lesion with complete capsule and two calcifications in the central part. **(B)** Sagittal image showed the mass adjacent to the sigmoid colon in the left iliac fossa.

A previously healthy 51-year-old man underwent a routine medical examination. An incidental pelvic solid mass was detected on ultrasonography (Figure 1) and computed tomography (CT) (Figure 2). The oval-shaped mass was about 50 × 40 mm in size and showed a low-density lesion with clear boundaries, a complete capsule, and two calcifications in the central part on the CT scan. The mass lay adjacent to the sigmoid colon in the left iliac fossa. The patient had no complaints or significant past medical history. No abnormality was found on physical exam including digital rectal examination. Tumor markers and other laboratory tests were within the normal range.

**Figure 3:**
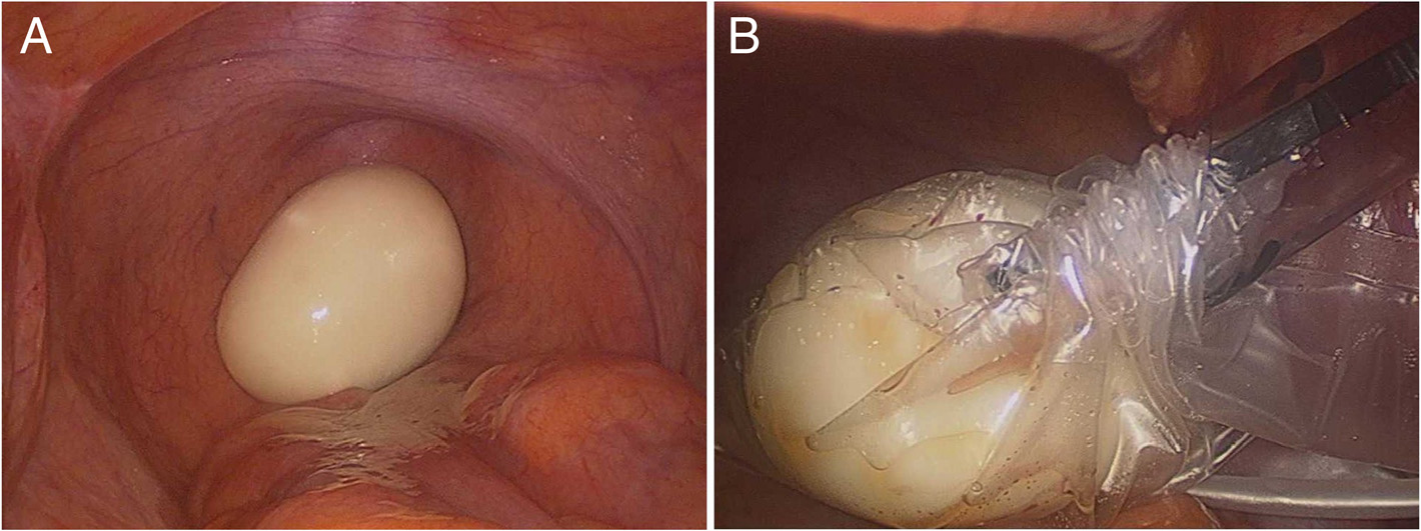
**Laparoscopic findings. (A)** A yellow-white, egg-shaped body that was completely free from the pelvic cavity was found in front of the rectum. **(B)** The body was put into an endoscopic retriever bag and taken out through the port site.

Based on the present imaging findings, the preoperative diagnosis of teratoma with core calcification or stromal tumor derived from the rectosigmoid colon was suspected. Accordingly, diagnostic laparoscopic surgery was performed. A yellow-white, egg-shaped body that was completely free from the pelvic cavity was found in front of the rectum (Figure 3A). Further laparoscopic exploration of pelvic and abdominal organs demonstrated that the liver, stomach, intestine, colon, and rectum were all normal. Finally, the peritoneal loose body was put into an endoscopic retriever bag, taken out through the enlarged port site in the right lower abdomen, and sent for histopathological examination (Figure 3B).

**Figure 4:**
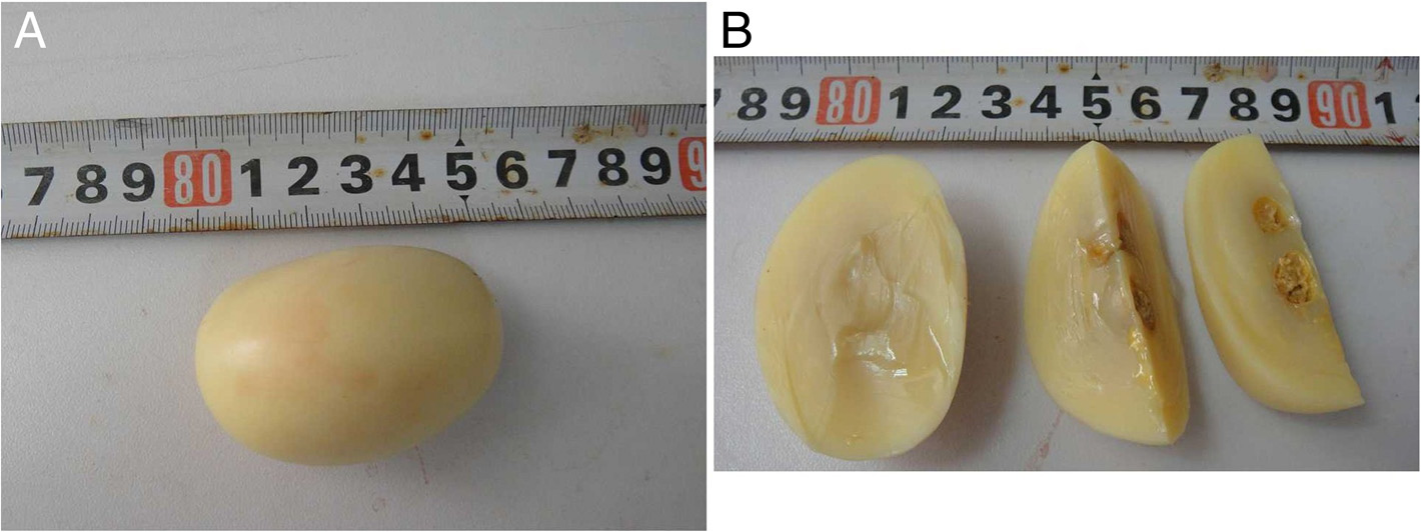
**Gross pathologic examination. (A)** The peritoneal loose body was 50 × 40 × 40 mm in size, oval-shaped, and yellow-white in appearance with a bony-hard, smooth surface, but without an obviously fibrous capsule. **(B)** The cross section displayed a thread-like appearance. There were two calcified cores filled with yellow cheese-like material.

**Figure 5:**
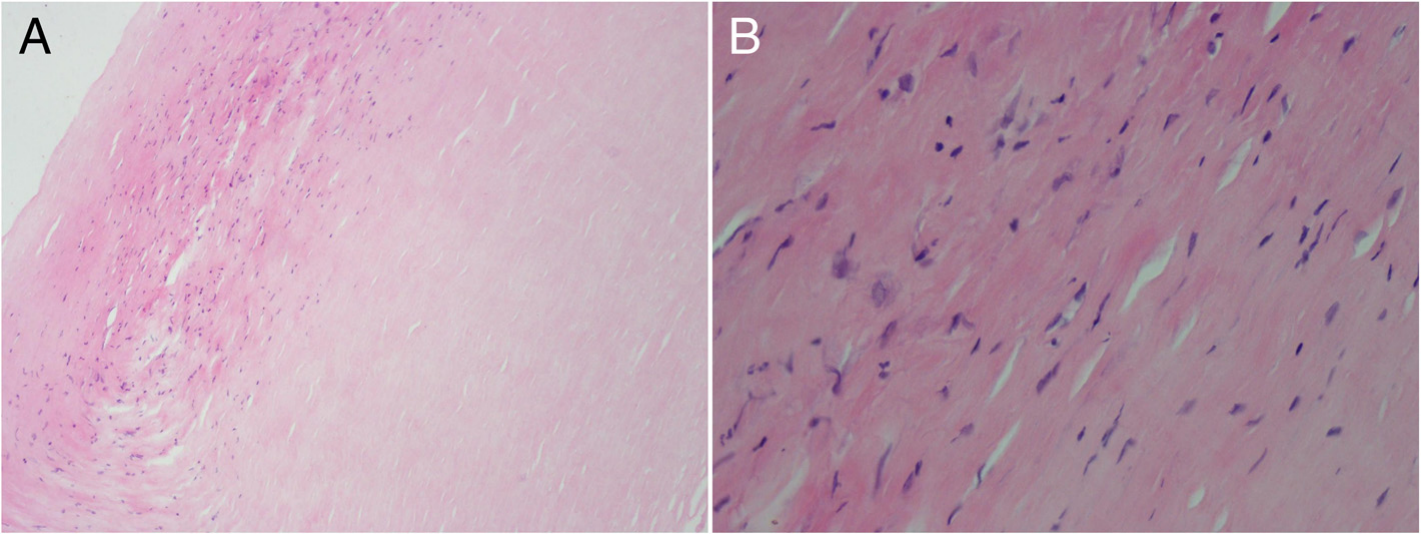
**Histologic findings. (A)** The lesion consisted of well-circumscribed, unencapsulated, paucicellular tissue, with an obviously hyalinized fibrosclerotic center [HE, ×100]. **(B)** At the periphery, the lesion was paucicellular, containing spindled fibroblasts embedded in a collagenous stroma. Scattered slit-like spaces were frequent [HE, ×400].

On gross pathologic examination, the peritoneal loose body measured 50 × 40 × 40 mm. It was yellow-white, oval in shape, and it had a bony-hard, smooth surface. The cross section displayed a thread-like appearance. There were two calcified cores filled with yellow cheese-like material, and the interval distance between the two cores was about 5 mm (Figure 4). Histologically, the lesion consisted of well-circumscribed, unencapsulated, paucicellular tissue, with an obviously hyalinized fibrosclerotic center. At the periphery, the lesion was paucicellular, containing spindled fibroblasts embedded in a collagenous stroma (Figure 5).

Our patient recovered well post-operatively. He was discharged from the hospital 2 days after surgery.

### Discussion

Peritoneal loose bodies are also called peritoneal mice. There is limited information about the incidence of peritoneal loose bodies around the world. They are very rare and usually incidentally diagnosed during surgery or autopsy. The characteristics of 22 cases that have been reported are shown in Table 1; we found that peritoneal loose body is more common in males. The incidence rate ratio between males and females is 18:4. The age span of patients at the time of diagnosis ranges from 2 months to 79 years, and the majority occurs in patients between 50 and 70 years old. Most peritoneal loose bodies range from 5 to 25 mm in size and generally do not cause any symptoms. When the maximum diameter reaches more than 50 mm, they can be called giant peritoneal loose bodies. The largest peritoneal loose body measured 95 × 86 mm and was reported by Mohri et al. [1] in 2007. Giant peritoneal loose bodies are not usually associated with specific symptoms except for chronic abdominal pain in some cases [1-3]. In our case, the giant peritoneal loose body was 50 × 40 × 40 mm in size and did not cause any discomfort; it was found incidentally on physical examination. Occasionally, if the peritoneal loose bodies are large enough and in a particular location, patients may be admitted to the hospital with acute urinary retention [4,5] or intestinal obstruction [6-8] due to extrinsic compression.

**Table 1:** **Summary of the information of 22 cases in the literature**

**Author**	**Published year**	**Gender**	**Age**	**Symptoms**	**Size of PLB (mm)**	**Weight of PLB (g)**	**Surgical methods**
Mohri et al. [1]	2007	M	73 years	Abdominal pain	95 × 75 × 66	220	Open
Hedawoo and Wagh [2]	2010	M	65 years	Abdominal pain	95 × 86	-	Open
Murat and Gettman [3]	2004	M	47 years	Pelvic pain	35 × 28 × 25	-	Laparoscopy
Bhandarwar et al. [4]	1996	M	65 years	Acute retention of urine	90 × 80	210	Open
Shepherd [5]	1951	M	79 years	Acute retention of urine	70 × 55	-	Open
Sewkani et al. [6]	2011	M	64 years	Abdominal pain	70 × 50	74	Open
Ghosh et al. [7]	2006	M	63 years	Intestinal obstruction	58 × 45 × 37 and 52 × 45 × 37	-	Open
Kao et al. [8]	2010	F	69 years	Intestinal obstruction	40 × 30 × 23	-	Open
Kogao et al. [10]	2010	F	33 years	Infertility	30 × 20	-	Laparoscopy
Gayer and Petrovitch [12]	2011	M	59 years	Incidental	30	-	Untreated
Nomura et al. [13]	2003	M	63 years	Incidental	50 × 40 × 30	-	Laparoscopy
Asabe et al. [14]	2005	F	2 months	Urinary tract infection	30	-	Laparoscopy
Kim et al. [15]	2013	M	50 years	Incidental	75 × 70 × 68	160	Laparoscopy
Sahadev and Nagappa [16]	2014	M	52 years	Abdominal pain	70 × 60	-	Laparoscopy
Jang et al. [17]	2012	M	60 years	Incidental	45 × 40 × 30	-	Laparoscopy
Nozu and Okumuta [18]	2012	M	67 years	Incidental	40	-	Untreated
Burns and James [19]	1969	F	33 years	Incidental	18 × 13	-	Open
Maekawa [20]	2013	M	58 years	Incidental	20	-	Open
Makineni et al. [21]	2014	M	52 years	Abdominal discomfort	60	-	Open
Allam et al. [22]	2013	M	77 years	Abdominal pain	17	-	Untreated
Huang et al. [23]	2011	M	55 years	Intestinal obstruction	-	-	Open
Takada et al. [24]	1998	M	79 years	Incidental	70 × 60 and 70 × 60	78 and 66	Open

PLB, peritoneal loose body.

Thus far, the exact pathogenesis of peritoneal loose bodies has not been clearly defined. Possible sources include: (1) appendix epiploica, (2) omentum [9], (3) autoamputated adnexa [10], or (4) fat tissue in the pancreas [11]. The most common source is appendix epiploica. It is believed that the process is sequential. First, chronic torsion of the appendix epiploica occurs, and the blood supply is shut off, followed by saponification and calcification of fat tissue. Finally, the appendix epiploica detaches from the colon due to atrophy of the pedicle and becomes a peritoneal loose body. Many authors suggest that the body gradually absorbs protein from peritoneal serum. The size of the peritoneal loose body increases slowly, like a snowball. However, the growth speed of the peritoneal loose body and the factors that promote or inhibit growth are unknown. Mohri et al. [1] discovered a peritoneal loose body in a 73-year-old man’s pelvic cavity that grew from 73 × 70 mm to 95 × 75 mm in 5 years. In addition, there was another case [12] of a peritoneal loose body that did not significantly change in size or appearance in 3 years. Interestingly, Koga K et al. [10] removed a 30 × 20 mm peritoneal loose body from a 33-year-old woman who, at 9 years of age, had adnexal torsion followed by calcification and autoamputation.

The differential diagnosis associated with peritoneal loose body include the following: (1) benign disease: leiomyoma, rhabdomyomas, teratoma, and fibroma; (2) malignant disease: colorectal cancer, ovarian cancer, and metastases; (3) calculous disease: urinary stones, gallstones, and appendix stones; (4) tubercular granuloma; and (5) others: calcification of lymph nodes, lymphoma, and foreign bodies. CT and MRI can be performed to distinguish peritoneal loose bodies from other lesions. For example, leiomyoma and some tumors enhance after injection of a contrast agent, while the appearance of peritoneal loose bodies remains unchanged.

Treatment is surgical removal because it is not easy to establish definite diagnosis preoperatively via physical examination and imaging technologies. Laparoscopic exploration is recommended [3,13-17]. Laparoscopy not only reduces surgical trauma but also shortens the patient’s hospitalization time. In our case, the patient was discharged from the hospital 2 days after surgery. Moreover, the loose body can be removed through a slightly enlarged trocar incision, and patients will not have a scar.

Until now, there have been no reports about the leading cause of death or recurrence in patients with peritoneal loose body. No harm has been shown to patients who receive active treatment.

## Conclusions

Peritoneal loose bodies are generally found incidentally. Clinically, if CT or other imaging shows an oval-shaped mass with or without calcifications in the central region, peritoneal loose body should be considered. Surgical removal is recommended for the patient with acute retention of urine or intestinal obstruction. Additionally, laparoscopy may be the best choice when the preoperative diagnosis is not clear and the lesion does not cause any clinical symptoms.

## Consent

Written informed consent was obtained from the patient for publication of this case report and any accompanying images. A copy of the written consent is available for review by the Editor-in-Chief of this journal.
